# Comparative analysis of lipid metabolism in trophoblast subpopulations in preeclampsia and *in vitro* hypoxia model

**DOI:** 10.3389/fmolb.2025.1731126

**Published:** 2025-12-09

**Authors:** Ivan Antipenko, Evgeny Knyazev, Timur Kulagin, Alexander Tonevitsky

**Affiliations:** 1 Faculty of Biology and Biotechnology, HSE University, Moscow, Russia; 2 Laboratory of Microfluidic Technologies for Biomedicine, Shemyakin-Ovchinnikov Institute of Bioorganic Chemistry of the Russian Academy of Sciences, Moscow, Russia

**Keywords:** preeclampsia, trophoblast, lipid metabolism, hypoxia, single-cell RNA-seq, SCARB1, PCSK9

## Abstract

Preeclampsia is a leading cause of maternal and perinatal morbidity associated with systemic lipid metabolism disturbances, yet the underlying molecular mechanisms remain incompletely understood. In this study, we integrated single-cell RNA-seq data from preeclamptic placentas with an in vitro hypoxia model to analyze gene expression changes across distinct trophoblast subpopulations. While all trophoblast lineages exhibited hypoxia-driven metabolic reprogramming, the response was highly cell-type specific. In the syncytiotrophoblast (SCT), the primary maternal-fetal barrier, preeclampsia was associated with a significant downregulation of LDLR and cholesterol biosynthesis genes (OR = 4.991, p = 6.30e−04). Concurrently, we observed increased expression of genes governing transcytosis (*SCARB1*, *CAV1*). In contrast, the extravillous trophoblast (EVT) displayed a divergent adaptive response, characterized by elevated LDLR expression and downregulated cholesterol biosynthesis. *In vitro* hypoxia modeling in BeWo b30 cells recapitulated the SCT-specific phenotype and identified a potential regulatory mechanism: a fivefold increase in *PCSK9* expression (padj = 3.53e−10) and a 1.5-fold decrease in *SNX17* (padj = 1.76e−04)—key regulators that limit lipoprotein receptor recycling. This was accompanied by the suppression of lipid biosynthesis genes and the transcriptional activation of pathways associated with transcytosis and cholesterol efflux. Collectively, these results confirm the pivotal role of hypoxic stress in disrupting placental lipid metabolism and reveal a subpopulation-specific transcriptional program in preeclampsia—a shift from endocytosis to transcytosis—that likely serves as a compensatory mechanism to ensure fetal lipid supply under conditions of limited availability.

## 1 Introduction

Preeclampsia represents one of the most severe complications of pregnancy, affecting 2%–8% of women worldwide (Dimitriadis et al., 2023; Ives et al., 2020). It accounts for up to 15% of maternal deaths (Say et al., 2014) and approximately 500,000 cases of intrauterine and neonatal mortality annually (Ngene and Moodley, 2024; Rana et al., 2019). This complex multisystem disorder is characterized by abnormal placentation in early pregnancy due to insufficient invasion of extravillous trophoblast (EVT) cells into the endometrium and inadequate remodeling of spiral arteries (Matsubara, 2017; Mitranovici et al., 2024). These processes lead to placental ischemia and hypoxia, which are traditionally considered key pathophysiological mechanisms of preeclampsia (Ives et al., 2020; Matsubara, 2017). The early-onset form of the disease (before 34 weeks of gestation) is marked by more pronounced defects in placentation and activation of hypoxic signaling pathways, including a significant increase in HIF-1α–mediated mechanisms (Matsubara, 2017; He et al., 2023).

The risk of developing preeclampsia is closely associated with lipid metabolism disorders. It increases with a higher proportion of trans-unsaturated fatty acids (TFAs) (Williams et al., 1998) and polyunsaturated fatty acids (PUFAs) (Clausen et al., 2001), with reduced serum omega-3 PUFA levels (Williams et al., 1995), as well as in the presence of maternal dyslipidemia (Wojcik-Baszko et al., 2018; Poornima et al., 2022). Moreover, lipid peroxidation products are elevated in preeclampsia and can induce endothelial damage in the mother (Hubel et al., 1989; Negre-Salvayre et al., 2022). Lipoprotein(a) [Lp(a)], the main carrier of oxidized phospholipids (Bergmark et al., 2008), has also been found to be significantly elevated in the plasma of preeclamptic patients (Bayhan et al., 2005; Konrad et al., 2020), which appears to correlate with disease development (Arifin et al., 2017). Metabolomic studies indicate that sterol, phospholipid, and sphingolipid metabolism is among the downregulated biochemical pathways in preeclampsia (Odenkirk et al., 2020; Yang et al., 2022; Hart, 2024). According to meta-analyses, women who later develop preeclampsia exhibit persistently elevated levels of low-density lipoproteins (LDL) throughout all trimesters, while high-density lipoproteins (HDL) decrease in the third trimester (Spracklen et al., 2014). Another meta-analysis demonstrated a significant increase in very-low-density lipoproteins (VLDL) before and during the onset of preeclampsia, implicating their role in endothelial dysfunction (Yang S. et al., 2025). Furthermore, pregnant women with preeclampsia show elevated maternal serum triglycerides, whereas umbilical cord blood displays increased cholesterol but not triglyceride levels (Stadler et al., 2023). These findings collectively confirm that alterations in maternal lipid metabolism play a crucial role in the pathogenesis of preeclampsia.

Pregnancy is accompanied by a pronounced remodeling of lipid metabolism. In the third trimester, lipolysis in adipose tissue intensifies, leading to elevated levels of free fatty acids, their re-esterification in the liver, and the development of maternal physiological hypercholesterolemia (Herrera et al., 2006; Jayalekshmi and Ramachandran, 2021). This condition is characterized by a 30%–50% increase in plasma lipoprotein concentrations during the second and third trimesters, followed by normalization after delivery (Wild and Feingold, 2023). Since the fetus has a limited capacity for endogenous lipid synthesis, the primary source of lipids is their transport from the maternal circulation across the placenta (Horne et al., 2019). Lipoproteins are unable to directly cross the placental barrier; therefore, their delivery occurs mainly through hydrolysis into free fatty acids at the cell surface, endocytosis of lipoprotein particles, and transcytosis, which ensures their direct transfer into the fetal bloodstream (Fuchs and Ellinger, 2004). Lipid transport through the trophoblast is mediated by receptor-dependent mechanisms (Cooke et al., 2021). The key mediators of this process include LDLR and SR-BI, which are involved in the uptake of low- and high-density lipoproteins (Kakava et al., 2022; Bolanle et al., 2025), as well as the ATP-binding cassette transporters ABCA1 and ABCG1, which regulate cholesterol efflux (Fuenzalida et al., 2020; Kallol and Albrecht, 2020). Collectively, these systems ensure both lipid uptake and transfer required for normal fetal development.

To investigate the mechanisms of nutrient transport and its regulation under hypoxic conditions, *in vitro* trophoblast cell models are commonly used (Knyazev et al., 2025). The BeWo b30 cell line is widely applied and is typically exposed to chemical hypoxia inducers such as oxyquinoline derivatives (OD), which act as inhibitors of HIF prolyl hydroxylases (Fuenzalida et al., 2022; Knyazev et al., 2019). These compounds mimic hypoxic conditions by stabilizing HIF-1α and activating the corresponding signaling cascades (Albers et al., 2019). However, the impact of hypoxia on lipid metabolism in such models and its correspondence to the alterations observed in preeclampsia *in vivo* remain insufficiently studied.

Cell lines provide a convenient means for modeling hypoxic conditions but fail to reproduce the heterogeneity of the trophoblast *in vivo*. The trophoblast is subdivided into villous cytotrophoblast (VCT), syncytiotrophoblast (SCT), and extravillous trophoblast (EVT), each subpopulation performing unique functions in placentation (Matsubara, 2017). Notably, the SCT serves as the main barrier and transport interface of the placenta (Cooke et al., 2021; Fan et al., 2024). Modern single-cell sequencing techniques allow for detailed analysis of molecular changes within these trophoblast subpopulations in preeclampsia, enabling the identification of specific gene expression patterns (Turco et al., 2018; Varberg et al., 2023).

The aim of this study was to compare the changes in the expression of lipid metabolism genes across three trophoblast subpopulations *in vivo* in preeclampsia with those observed in the BeWo b30 cell line *in vitro* under chemically induced hypoxia. The study design is presented in the Supplementary Figure S1. The obtained data made it possible to identify differences in lipid metabolism among distinct trophoblast subpopulations and to characterize the alterations occurring in preeclampsia and under induced hypoxia *in vitro*, which is essential for the validation and application of existing cellular models of the disease.

## 2 Materials and methods

### 2.1 Single-cell mRNA-seq analysis

Single-cell mRNA-seq analysis was performed using previously published data by Admati et al. (2023). Briefly, the dataset included samples from early-onset preeclampsia, with a total of 10 preeclampsia cases compared to three non-preeclamptic controls, all from primiparous singleton pregnancies. All donors were diagnosed according to the American College of Obstetricians and Gynecologists criteria (Gynecologists, 2020). Exclusion criteria included chronic maternal diseases and fetal malformations.

Cell clusters were visualized using principal component analysis (PCA) and UMAP based on scRNA-seq data preprocessed according to standard procedures using the Scanpy package, version 1.11.4 (Wolf et al., 2018; McInnes et al., 2025). Differential gene expression analysis within the main trophoblast cell clusters (VCT, SCT, EVT) was conducted using the non-parametric Mann–Whitney U test with multiple-testing correction according to the Benjamini–Hochberg method.

Additionally, normalized counts per million (CPM) were calculated from the raw data. For each cluster–experimental group combination, the fraction of cells with detectable expression, the median, and the 95th percentile of expression levels were computed. For further analysis of gene expression intensity, a subset of cells with expression above the global 95th percentile CPM within each cluster was selected, and expression values were compared between groups using analysis of variance (ANOVA) from the SciPy package, version 1.16.2 (Virtanen et al., 2020).

### 2.2 Differential gene expression analysis in trophoblast populations

To further assess differential gene expression (DEGs), a pseudobulk analysis was performed. For this, raw counts were aggregated within each biological sample and cell type, followed by differential expression analysis using the DESeq2 package, version 1.48.2 (Love et al., 2014). Based on the resulting data, pathway enrichment analysis (GSEA) was conducted using the fgsea package, version 1.34.2, against the Hallmark and Gene Ontology: Biological Process (GO:BP) collections, with Wald test statistics from DESeq2 used as the ranking metric (Subramanian et al., 2005; Korotkevich et al., 2016; Liberzon et al., 2015; Ashburner et al., 2000). Statistical significance of overlaps between DEG sets was evaluated using Fisher’s exact test.

### 2.3 Cell culture

BeWo b30 choriocarcinoma cells, used as a model of trophoblasts, were kindly provided by Prof. Dr. Christiane Albrecht (University of Bern, Switzerland) with permission from Prof. Dr. Alan Schwartz (Washington University in St. Louis, United States). BeWo b30 cells were cultured in 25 cm^2^ flasks with ventilated caps (Costar, Corning) at 37 °C and 5% CO_2_ in an MCO-18AC incubator (Sanyo), in DMEM medium (4.5 g/L glucose) supplemented with 10% FBS (Capricorn, United States), 1% MEM Non-Essential Amino Acids (100×), 2 mM L-glutamine, and 1% Penicillin–Streptomycin solution (10,000 U/mL) (all reagents from Gibco, Thermo Fisher Scientific, United States). Cells were passaged every 2–3 days using 0.25% trypsin–EDTA solution (PanEco, Cat. No. P034). Cell count, viability, and size were assessed by trypan blue staining using a LUNA-II Automated Cell Counter (Logos Biosystems).

### 2.4 Hypoxia induction in BeWo b30 cells

For hypoxia induction experiments, once BeWo b30 cells reached approximately 80% confluence, the culture medium was replaced either with fresh medium (control) or with medium containing 5 µM of the 8-hydroxyquinoline derivative 4,896–3,212 (ChemRar, Russia), a chemical activator of the HIF-1 signaling pathway (Knyazev et al., 2021). In all experimental conditions, the medium contained 0.05% DMSO. After 24 h of incubation, cells were lysed in 700 µL of QIAzol Lysis Reagent (QIAGEN, Germany).

### 2.5 mRNA sequencing

RNA preparation was performed as previously described (Knyazev et al., 2025). Briefly, total RNA was extracted using the miRNeasy Mini Kit (QIAGEN, Germany). RNA concentration was determined using a NanoDrop 1,000 spectrophotometer (Thermo Fisher Scientific, United States), and RNA quality was assessed with an Agilent 2,100 Bioanalyzer (Agilent Technologies, United States). The RNA integrity number (RIN) was at least nine for all analyzed samples.

Next-generation sequencing (NGS) libraries were prepared using the Illumina Stranded mRNA Library Prep Kit (Illumina, United States) to generate 75-bp single-end reads. Sequencing was performed on a NextSeq 550 system (Illumina, United States).

### 2.6 mRNA sequencing data analysis

Quality assessment of the obtained FASTQ files was performed using FastQC v0.11.9 (Simon, 2010). Adapter sequences and low-quality bases were trimmed with cutadapt v2.10 (Martin, 2011). The processed reads were mapped to the human reference genome (GENCODE GRCh38. p13) using the STAR v2.7.5b aligner (Dobin et al., 2013).

Library size normalization was carried out using the trimmed mean of M-values (TMM) method implemented in the edgeR v3.30.3 package (Chen et al., 2025), with default filtering of lowly expressed genes. Normalized FPKM (fragments per kilobase of transcript per million mapped reads) values were calculated using the same package and log-transformed. For subsequent analyses, only highly expressed genes were retained, excluding the 25% of genes with the lowest median FPKM values in each experimental group. The obtained results have been deposited in the Gene Expression Omnibus (GEO) under accession number GSE308908.

To evaluate changes in cellular signaling pathway activity after hypoxia induction in BeWo b30 cells, Gene Set Enrichment Analysis (GSEA) was performed using the MSigDB Hallmark gene set collection. Genes were ranked according to the Wald statistic obtained from differential expression analysis using DESeq2 (Love et al., 2014). Enrichment significance was determined based on the normalized enrichment score (NES) and FDR q-value (<0.05).

### 2.7 Bioinformatic analysis of transcription factor activity

To assess the statistical significance of transcription factor activation changes, a one-sided Fisher’s exact test (fisher_exact, SciPy library v1.13.1) was applied. The test was conducted on a list of known transcription factor targets obtained from the TRRUST v.2 database (https://www.grnpedia.org/trrust/) (Han et al., 2018), which also provided the direction of regulation. Transcription factor activation was determined based on changes in the expression of target genes consistent with the direction of regulation. For Activation, an increase in expression with log_2_FC greater than 0.6 (FDR <0.05) was considered. For Repression, a decrease in expression with log_2_FC less than −0.6 (FDR <0.05) was taken into account, compared to the control cell line. Transcription factors were considered significantly enriched in activated targets if the Fisher’s exact test FDR value, adjusted by the Benjamini–Hochberg method, was <0.05.

### 2.8 Real-time PCR analysis

Cells were lysed using QIAzol Lysis Reagent (Qiagen, Cat. No. 79306), and total RNA was extracted with the miRNeasy Mini Kit (Qiagen, Cat. No. 74104) according to the manufacturer’s protocol. RNA concentration was measured using a NanoDrop ND-1000 spectrophotometer (Thermo Fisher Scientific, United States). Reverse transcription was performed using the MMLV Reverse Transcriptase Kit (Eurogene, Cat. No. SK021) following the manufacturer’s instructions, and the resulting complementary DNA (cDNA) was stored at −20 °C. Quantitative PCR (qPCR) was carried out on a DT-96 thermocycler (DNA-Technology, Russia) using the qPCRmix-HS SYBR reagent (Eurogene, Cat. No. PK147L) according to the manufacturer’s recommendations. Oligonucleotide primers for qPCR were designed based on mRNA sequences obtained from the UCSC Genome Browser database (Karolchik et al., 2003) using the Primer-BLAST tool (Ye et al., 2012). Potential secondary structure formation and homo-/heterodimerization were evaluated using OligoAnalyzer 3.1 (https://www.idtdna.com/pages/tools/oligoanalyzer). The reference genes *ACTB* and *GUSB* were used for normalization. Primer sequences are provided in Supplementary Table S1.

### 2.9 Lipid droplet staining

BeWo b30 cells were seeded into 96-well plates at a density of 3 × 10^4^ cells per well (in five replicates) and incubated overnight. The next day, cells were treated with 5 µM of the 8-hydroxyquinoline derivative 4,896–3,212 for 24 h. Control cells were cultured in parallel wells with the addition of 0.05% DMSO to the culture medium. For Oil Red O staining of lipid droplets, cells were washed twice with PBS, fixed in 10% formalin for 30 min, washed twice with distilled water (1 min each), and incubated for 5 min in 60% isopropanol. Cells were then stained with Oil Red O solution (1.8 mg/mL in 60% isopropanol) for 20 min, followed by five washes with deionized water to remove excess dye. Stained cells were visualized using a ZOE Fluorescent Cell Imager (Bio-Rad, United States). For quantitative analysis of lipid accumulation, the retained Oil Red O dye was extracted with 100% isopropanol under gentle shaking at room temperature for 5 min. The extracts were transferred to a new 96-well plate, and optical density was measured at 492 nm using a SpectraMax i3x microplate reader (Molecular Devices, United States). Statistical analysis was performed using the Mann–Whitney U test.

### 2.10 Exosome isolation and nanoparticle tracking analysis (NTA)

For hypoxia induction, BeWo b30 cells were incubated either in the presence of the 8-hydroxyquinoline derivative 4,896–3,212 (OD) or, under control conditions, with the addition of 0.05% DMSO. After 24 h, the culture medium was replaced with serum-free medium, and the cells were incubated for an additional 24 h. The collected conditioned medium was filtered through a 0.22 µm syringe filter (Pall Life Sciences, Port Washington, United States). A PEG 6000 solution (Sigma-Aldrich, St. Louis, United States) was added to the filtrate to a final concentration of 8% (w/v), and the mixture was incubated at 4 °C for 16 h. Samples were then centrifuged at 16,000 × g for 1 h at 4 °C. The resulting small extracellular vesicle (sEV) pellet was washed with PBS, resuspended in 0.2 mL of filtered PBS, and immediately analyzed by Nanoparticle Tracking Analysis (NTA).

Particle size distribution and concentration of isolated vesicles were determined using Nanoparticle Tracking Analysis (NTA) on a NanoSight NS300 instrument (NanoSight Ltd., Amesbury, UK). All measurements were performed in accordance with ASTM E2834-12R18 guidelines. Samples were diluted with filtered PBS to a final concentration of approximately 1.5 × 10^8^ particles/mL. Videos of Brownian motion were recorded at room temperature with passive temperature sensing under the following settings optimized for EV analysis: camera level 12, shutter 850, gain 450, low threshold 715, high threshold 10,725. Video processing was performed using NTA software version 3.4 build 0,033 (NanoSight Ltd.) with a detection threshold of 5%. For reliable quantification, at least five 60-s videos were recorded and analyzed at 25 frames per second. Data from multiple videos were combined to generate a particle size distribution histogram and to calculate the mean total particle concentration, accounting for the dilution factor. Statistical analysis of differences between the control group and the group treated with the 8-hydroxyquinoline derivative was performed using one-way ANOVA.

## 3 Results

### 3.1 Identification and characterization of trophoblast subpopulations

**FIGURE 1:**
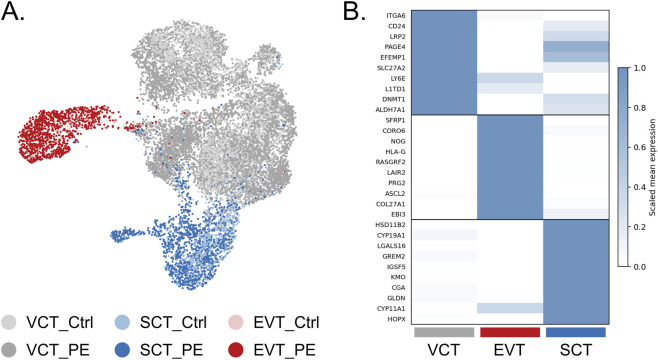
Identification and Characterization of Trophoblast Subpopulations. **(A)** UMAP clustering of trophoblast cells based on single-cell RNA-seq data. VCT (cytotrophoblast) cells are shown in gray, SCT (syncytiotrophoblast) in blue, and EVT (extravillous trophoblast) in red; **(B)** Heatmap showing the top 10 most differentially expressed genes characteristic of each trophoblast population.

Previously published single-cell RNA-sequencing data were used for analysis. From the complete dataset, trophoblast cells were extracted and subdivided into three subpopulations: syncytiotrophoblast (SCT, n = 2,104), extravillous trophoblast (EVT, n = 1,238), and villous cytotrophoblast (VCT, n = 9,967) (Figure 1A).

The thirty most significant differentially expressed genes among trophoblast subtypes are shown in the heatmap (Figure 1B). Several of these correspond to well-established markers of specific trophoblast lineages: *PRG2* and *EBI3* for EVT; *CGA* and *CYP19A1* for SCT; *PAGE4* and *ITGA6* for VCT (Zhou et al., 2022; Hoshiyama et al., 2025; Zhang et al., 2025).

### 3.2 Differential expression and gene signature changes in trophoblast populations

**FIGURE 2:**
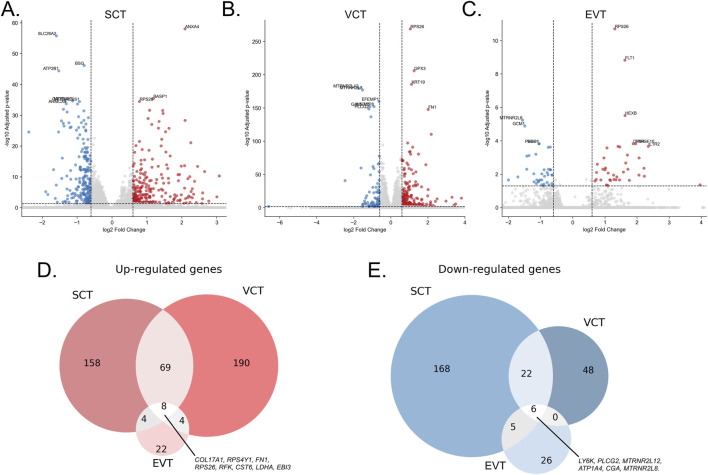
Differential gene expression in trophoblast populations in preeclampsia compared to controls (|Log2FC| > 0.6, padj < 0.05). **(A)** Volcano plot for SCT; **(B)** Volcano plot for VCT; **(C)** Volcano plot for EVT; **(D)** Venn diagram of upregulated genes shared between trophoblast populations; **(E)** Venn diagram of downregulated genes.

Analysis of differential gene expression between trophoblast populations in preeclampsia and control samples revealed that the highest number of genes with |log_2_FC| > 0.6 and *padj* < 0.05 was detected in SCT (440 genes), while the lowest number was observed in EVT (75 genes) (Figures 2A–C).

Eight genes were upregulated in preeclampsia across all trophoblast populations (Figure 2D). Among them, fibronectin 1 (*FN1*), a marker of vascular injury previously reported to be elevated in preeclamptic patients (Wu et al., 2021), and lactate dehydrogenase A (*LDHA*), whose serum levels have been suggested as a marker of preeclampsia and disease severity (Nasir et al., 2025).

Conversely, six genes were downregulated across all clusters (Figure 2E), including *CGA* (Glycoprotein Hormones, Alpha Polypeptide), which encodes the α-subunit of several glycoprotein hormones, including chorionic gonadotropin (hCG), luteinizing hormone (LH), follicle-stimulating hormone (FSH), and thyroid-stimulating hormone (TSH). During pregnancy, its role in hCG synthesis is crucial, as hCG maintains corpus luteum function and stimulates progesterone production. Previous studies have shown that reduced hCG expression may be associated with impaired placental function in early pregnancy (Tóth et al., 2024), including in preeclampsia (Mohammed et al., 2022).

Gene set enrichment analysis (GSEA) using the MSigDB HALLMARK collection revealed differences between trophoblast populations: EVT showed 22 upregulated and nine downregulated pathways, SCT had nine upregulated and six downregulated, and VCT displayed six upregulated and five downregulated pathways (see Supplementary Figure S2). Among the pathways significantly upregulated across all clusters were *HALLMARK_HYPOXIA*, *HALLMARK_ESTROGEN_RESPONSE_EARLY, HALLMARK_INTERFERON_ALPHA_RESPONSE, HALLMARK_TNFA_SIGNALING_VIA_NFKB,* and *HALLMARK_INTERFERON_GAMMA_RESPONSE*. Downregulated pathways common to all populations included *HALLMARK_G2M_CHECKPOINT, HALLMARK_OXIDATIVE_PHOSPHORYLATION, HALLMARK_ADIPOGENESIS, and HALLMARK_E2F_TARGETS*. Notably, *HYPOXIA* was among the most strongly activated pathways in all populations: EVT–NES = 2.34 (*padj* = 6.30e-11); SCT–NES = 2.16 (*padj* = 2.17e-9); VCT–NES = 1.87 (*padj* = 1.40e-5).

Analysis of MSigDB GO: Biological Process pathways indicated that preeclampsia affects multiple lipid metabolism-related pathways. In EVT, the *GOBP_MEMBRANE_LIPID_CATABOLIC_PROCESS* pathway was upregulated (NES = 1.98, *padj* = 0.011), suggesting increased demand for energy or membrane components. In SCT, *GOBP_FATTY_ACYL_COA_METABOLIC_PROCESS* was significantly downregulated (NES = −2.08, *padj* = 0.035), indicating reduced fatty acid (CoA-derivative) metabolism, which may decrease fatty acid oxidation and lipid-based energy production. In VCT, *GOBP_MEMBRANE_LIPID_METABOLIC_PROCESS* was also downregulated (NES = −1.56, *padj* = 0.024). Collectively, these results suggest that preeclampsia activates lipid catabolic processes in EVT, whereas SCT and VCT exhibit suppressed lipid metabolism, reflecting differences in metabolic demands and functional states among trophoblast subpopulations.

### 3.3 Analysis of metabolic gene expression in trophoblast populations

Lipoproteins are internalized into trophoblast cells via both clathrin- and caveolin-dependent endocytic pathways (Cooke et al., 2021). For LDL, the primary mechanism is clathrin-mediated endocytosis through the LDL receptor (*LDLR*), in which the adaptor protein ARH (*LDLRAP1*) links the receptor to the clathrin complex (Maxfield and van Meer, 2010; Go and Mani, 2012; Palinski, 2009; He et al., 2002). After internalization, *LDLR* is recycled back to the cell surface with the involvement of Sorting nexin 17 (*SNX17*) (Farfán et al., 2013; Burden et al., 2004). Proprotein convertase subtilisin/kexin type 9 (*PCSK9*) plays a key regulatory role by directing LDLR to lysosomal degradation (Zhang et al., 2008). In addition to LDLR, SR-BI (*SCARB1*) participates in lipoprotein endocytosis; it can bind both LDL and HDL (Wittmaack et al., 1995; Wadsack et al., 2003). Unlike LDLR, SR-BI undergoes caveolin-mediated internalization (Kakava et al., 2022) and facilitates transcytosis of captured lipoproteins (Bolanle et al., 2025).

**FIGURE 3:**
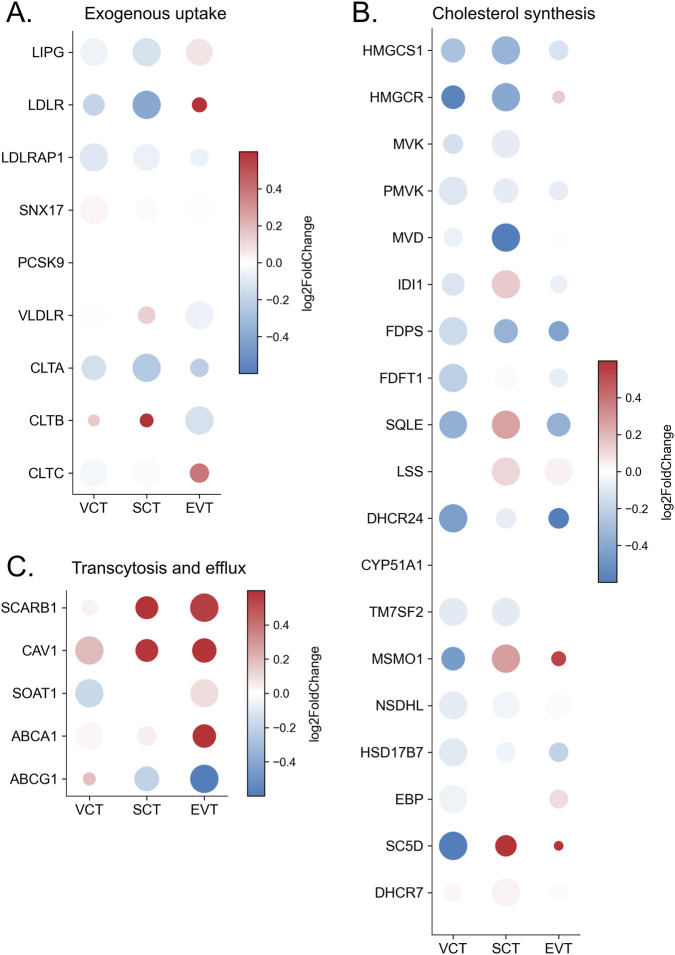
Dot plot of gene expression changes in trophoblast populations (VCT, SCT, EVT) in preeclampsia relative to control. **(A)** Genes involved in exogenous lipid uptake. **(B)** Cholesterol biosynthesis genes. **(C)** Genes involved in lipid transcytosis and efflux. Dot color indicates log2 fold change of the 95th percentile CPM (red–upregulated, blue–downregulated, white–unchanged). Dot size reflects the relative expression level of each gene in the control group (95th percentile CPM).

Analysis of single-cell sequencing data from trophoblast subpopulations revealed that genes involved in exogenous cholesterol uptake showed the most pronounced changes for LDLR and clathrin light chains (*CLTA*, *CLTB*) (Figure 3). Among trophoblast populations, *LDLR* expression was highest in the SCT cluster (Figure 3), consistent with the role of SCT in maternal lipoprotein uptake (Bolanle et al., 2025). In preeclampsia, *LDLR* expression decreased in VCT and SCT, with the most pronounced reduction observed in SCT (95th percentile CPM: Ctrl = 608.6; PE = 462.1, p = 0.025) (Supplementary Figure S3A). Conversely, in EVT, a trophoblast population with initially low LDLR expression, its levels increased, both in the 95th percentile CPM (Ctrl = 181.6; PE = 380.6, p = 0.037) and in median CPM and the percentage of positive cells (Supplementary Figure S3).

Additionally, *CLTA* expression decreased across all clusters, whereas *CLTB* expression increased in SCT (95th percentile CPM: Ctrl = 627; PE = 946, p = 3.55e-06). Considering that clathrin heavy chain (*CLTC*) is initially expressed at a lower level, it may act as a limiting factor in clathrin-mediated endocytosis. *CLTC* expression remained stable in VCT and SCT and showed a non-significant increase in EVT (95th percentile CPM: Ctrl = 276.9; PE = 363.3; p = 0.79).

*De novo* cholesterol biosynthesis is a series of sequential reactions predominantly occurring on the endoplasmic reticulum membrane (Ikonen, 2008). Analysis of gene expression involved in cholesterol biosynthesis across trophoblast populations showed higher expression in VCT and SCT compared to EVT (Figure 3B; Supplementary Figure S4).

Preeclampsia led to reduced expression of most cholesterol biosynthesis genes in all trophoblast populations. In VCT, 16 out of 19 genes were downregulated, including the rate-limiting enzyme HMGCR (*p* = 7.82e-07) (Ridgway and McLeod, 2008). In SCT, 11 out of 19 genes exhibited decreased expression, also including *HMGCR* (95th percentile CPM: Ctrl = 383.8; PE = 291.6, *p* = 0.0036). In EVT, 10 out of 19 cholesterol biosynthesis genes showed reduced expression in preeclampsia (Figure 3B).

To assess the statistical significance of these changes in the context of global transcriptomic alterations, we tested the hypothesis that cholesterol biosynthesis gene downregulation is significant. In VCT, 7,323 out of 24,859 genes showed lower expression in preeclampsia compared to control, confirming the significance of cholesterol biosynthesis gene downregulation (odds ratio, OR = 12.8; *p* = 1.17e-06). Statistically significant results were also observed in SCT (4,536 out of 20,970 genes downregulated; OR = 4.991; *p* = 6.30e-04) and EVT (5,181 out of 20,459 genes downregulated; OR = 4.061; *p* = 2.55e-03).

*SCARB1*, encoding the main receptor mediating lipoprotein particle transcytosis, showed increased expression in preeclampsia in SCT (95th percentile CPM: Ctrl = 176.7; PE = 271.5, *p* = 3.00e-07) and EVT (95th percentile CPM: Ctrl = 265.1; PE = 389.2, *p* = 0.037) clusters. In addition, *CAV1*, required for SCARB1-mediated caveolin-dependent endocytosis, was upregulated in all clusters (*p* < 0.01) (Figure 3C).

Interestingly, *SOAT1*, encoding the enzyme catalyzing cholesterol esterification and subsequent storage in lipid droplets, was not expressed in SCT. The absence of *SOAT1* may reflect a barrier function of this cell population rather than a storage role.

In EVT, preeclampsia induced pronounced changes in cholesterol transporter expression: *ABCG1* was significantly downregulated (95th percentile CPM: Ctrl = 335.2; PE = 146.0, *p* = 3.60e-07), while *ABCA1* showed a trend toward upregulation (95th percentile CPM: Ctrl = 261.4; PE = 528.2, *p* = 0.11) (Figure 3C; Supplementary Figure S5).

### 3.4 Analysis of differentially expressed genes in BeWo b30 cells following hypoxia induction

**FIGURE 4:**
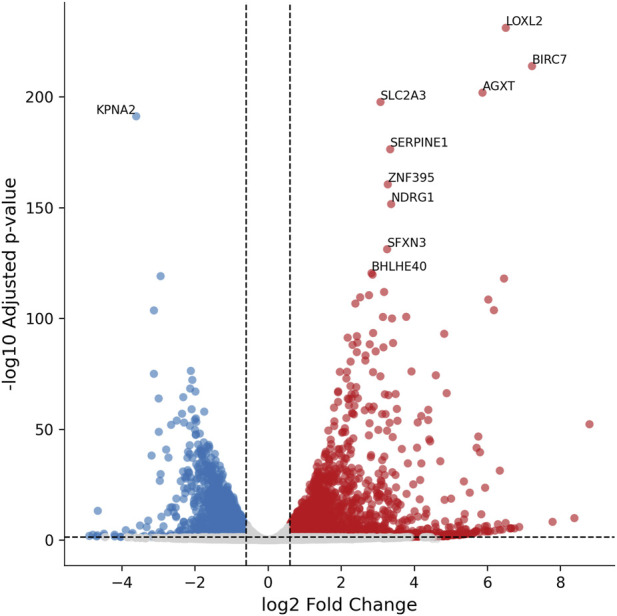
Volcano plots illustrating differential gene expression in BeWo b30 cells following hypoxia induction by OD treatment. Thresholds: |log2FC| > 0.6, FDR-adjusted p-value <0.05, baseMean >1.

Hypoxia induction using the oxoquinoline derivative in BeWo b30 cells resulted in changes in the expression of 4,879 genes, of which 2,562 were upregulated and 2,317 were downregulated (see Figure 4). The full list of genes is provided in Supplementary Table S2.

Among the most significantly altered genes following OD treatment were *LOXL2* (log2FC = 6.51, padj = 6.79e-232), *BIRC7* (log2FC = 7.22, padj = 1.29e-214), *KPNA2*, and *BHLHE40*. These genes are associated with hypoxic signal transduction, epithelial–mesenchymal transition (EMT), and cellular adaptation to stress. *LOXL2*, a known HIF target, participates in extracellular matrix remodeling under hypoxic conditions (Schietke et al., 2010), whereas *BHLHE40* (DEC1), induced by HIF-1α, acts as a transcriptional repressor of angiogenesis and proliferation (Acosta-Iborra et al., 2024). In addition, a pronounced upregulation of *SLC2A3* (GLUT3, log2FC = 3.08, padj = 2.03e-198) — a glucose transporter—is consistent with its well-established role as a hypoxia marker in trophoblast cells (Hu et al., 2022).

Analysis of overlapping differentially expressed genes (DEGs) in BeWo b30 cells revealed the strongest concordance with syncytiotrophoblasts (SCT) after preeclampsia: 171 shared genes, including 119 unique to the BeWo b30 Oxy–SCT intersection. The number of overlapping genes with villous cytotrophoblasts (VCT) and extravillous trophoblasts (EVT) was 124 and 26, respectively (see Supplementary Figure S6A). Comparison with OD-treated cells demonstrated a pronounced concordance of DEGs between BeWo b30 and SCT (OR = 4.46; n = 391; padj = 5.4e-12). In VCT, the effect was weak and not statistically significant (OR = 1.18; n = 935; padj = 0.17), while in EVT, the odds ratio was high but did not reach significance due to the small overlap (OR = 5.00; n = 32; padj = 0.065).

Furthermore, OD treatment in BeWo b30 cells led to increased expression of *FN1* (FC = 1.45, padj = 0.045) and *LDHA* (FC = 1.64, padj = 1.04e-08), along with decreased expression of *CGA* (FC = −1.41, padj = 0.0018), which mirrors the expression changes observed across all trophoblast subpopulations in preeclampsia. These findings highlight the similarity between OD-induced hypoxia in BeWo cells and syncytiotrophoblasts in preeclampsia (Zhao et al., 2021), further supported by the expression of *CGA* and *CYP19A1* markers (see Supplementary Figure S6B).

### 3.5 Changes in signaling pathways during the hypoxic response in BeWo b30 cells

To evaluate the biological processes involved in the BeWo b30 cell response to hypoxia induction, a GSEA enrichment analysis was performed using the HALLMARK gene set collection. A total of 32 significantly enriched signatures were identified, including 16 upregulated and 16 downregulated pathways (Supplementary Table S3). Pathways that showed consistent upregulation both in trophoblast populations affected by preeclampsia and in BeWo b30 cells under OD-induced hypoxia included *HALLMARK_HYPOXIA* (NES = 2.73, padj = 2.31e-30), *HALLMARK_TNFA_SIGNALING_VIA_NFKB* (NES = 2.21, padj = 1.47e-11), and *HALLMARK_ESTROGEN_RESPONSE_EARLY* (NES = 1.57, padj = 0.0008). In contrast, pathways exhibiting decreased activity included *HALLMARK_G2M_CHECKPOINT* (NES = −2.54, padj = 4.35e-26), *HALLMARK_OXIDATIVE_PHOSPHORYLATION* (NES = −2.52, padj = 7.61e-25), *HALLMARK_E2F_TARGETS* (NES = −2.58, padj = 4.86e-29), and *HALLMARK_ADIPOGENESIS* (NES = −1.55, padj = 0.0017).

To assess independent activation or repression of pathways, overlap analysis of genes from the GSEA leading edges was performed and visualized as interaction graphs. Among the upregulated gene sets, a single major cluster was formed, centered around Hypoxia, Glycolysis, and TNFα signaling via NFκB. For example, within the Glycolysis pathway, 58 out of 189 genes were part of the leading edge, 36 of which (62%) overlapped with Hypoxia (Supplementary Figure S7). The high degree of clustering, reflecting a substantial overlap of activated genes, indicates partial dependence of these pathways on the hypoxic response, which appears to serve as a central regulatory core.

Among the downregulated gene sets in BeWo b30 cells after OD treatment, two major clusters were identified (Supplementary Figure S8). The first cluster comprised G2M checkpoint and E2F targets (with 57/133 (43%) and 136/324 (42%) leading-edge gene overlap, respectively). The second cluster was centered around Oxidative phosphorylation, showing overlap with Fatty acid metabolism (15/42, 36%) and Adipogenesis (26/70, 37%). These findings suggest partially independent suppression of these pathways, reflecting a simultaneous inhibition of metabolism and cell proliferation.

A hypergeometric analysis of transcription factor activity revealed two functional groups of significantly activated regulators (Supplementary Table S4). The first group (HIF1A, NFKB1, TP53) was associated with hypoxic and cellular stress responses, reflecting the activation of oxygen deprivation–related mechanisms. The second group (WT1, JUN, KLF4) was linked to vascular differentiation and trophoblast function, indicating an influence on placental development and vascular processes. Among the downregulated transcription factors were E2F1 (padj = 0.0036), which controls the cell cycle and proliferation, and MYC (padj = 0.0094), a key regulator of cell growth and metabolism, including lipid metabolism. Together with the inactivation of the corresponding signaling pathways (Supplementary Table S2), these results indicate a global suppression of metabolic and proliferative activity in BeWo b30 cells under OD-induced hypoxia.

### 3.6 Changes in the expression of lipid metabolism genes induced by hypoxia in BeWo b30 cells

**FIGURE 5:**
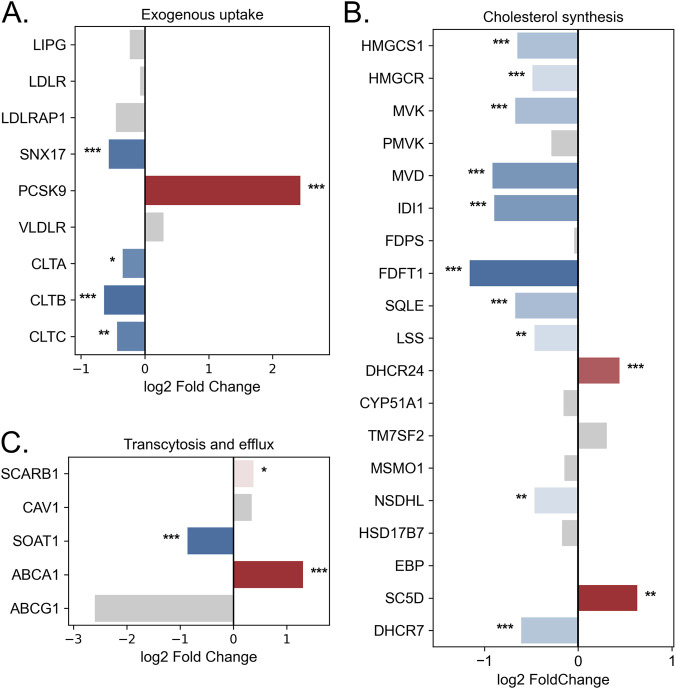
Changes in the expression of lipid metabolism genes in BeWo b30 cells following OD treatment. **(A)** Genes involved in exogenous lipid uptake. **(B)** Genes of cholesterol biosynthesis. **(C)** Genes involved in lipid transcytosis and efflux. Bar color represents log2 fold change (blue—downregulation, red—upregulation, gray—non-significant changes). Asterisks indicate statistical significance (*padj*: p < 0.05 — *, p < 0.01 — **, p < 0.001 — ***).

Based on transcriptomic analysis, *LDLR* expression did not change significantly (FC = −1.05, padj = 6.86e-01) (Figure 5A), which was consistent with the qPCR results (FC = 1.469, p = 0.18). In contrast, OD treatment caused a significant downregulation of *CLTA* (FC = −1.27, padj = 2.83e-02), *CLTB* (FC = −1.56, padj = 2.55e-05), and *CLTC* (FC = −1.35, padj = 1.99e-03; qPCR: FC = −2.27, p = 0.0071). At the same time, OD induced a marked increase in *PCSK9* expression by 5.41-fold (padj = 3.53e-10) and a 1.5-fold decrease in *SNX17* expression (padj = 1.76e-04), further reducing the efficiency of exogenous cholesterol uptake. Collectively, these findings indicate that exogenous cholesterol uptake is impaired under OD-induced hypoxia in BeWo b30 cells.

Hypoxia induction in BeWo b30 cells led to a decrease in the expression of genes involved in cholesterol biosynthesis. OD treatment reduced the expression of 10 out of 19 genes in this pathway, including *HMGCS1*, which catalyzes the first step of cholesterol synthesis (FC = −1.57, padj = 2.85e-06; qPCR: FC = −2.30, p = 0.036), and the rate-limiting enzyme *HMGCR* (FC = −1.40, padj = 3.76e-04; qPCR: FC = −2.54, p = 0.030) (Figure 5B). At the same time, expression of *SC5D* and *DHCR24* increased. Fisher’s exact test indicated that OD-induced hypoxia caused a statistically significant suppression of the cholesterol biosynthesis pathway compared with global transcriptomic changes (OR = 4.22, p = 2.21e-03).

Changes were also observed in genes related to lipid transport to the fetus. A trend toward increased *SCARB1* expression was noted (FC = 1.30, padj = 1.39e-02; qPCR: FC = 2.24, p = 0.13), along with a significant upregulation of the cholesterol exporter *ABCA1* (2.5-fold; padj = 2.64e-06) (Figure 5C). Concurrently, *SOAT1* expression decreased (FC = −1.82, padj = 1.03e-09; qPCR: FC = −2.61, p = 0.025), indicating reduced cholesterol esterification and lipid droplet formation.

Additionally, the diacylglycerol O-acyltransferases *DGAT1* and *DGAT2*, which catalyze the final steps of triglyceride synthesis (covalent attachment of acyl-CoA to diacylglycerol) and contribute to lipid droplet formation (Zadoorian et al., 2023), were affected. OD treatment reduced *DGAT1* expression by 1.65-fold (p = 0.00016) and *DGAT2* by 1.64-fold (p = 0.63; not statistically significant). Lipid droplet accumulation was further assessed using Oil Red O staining (Supplementary Figure S7). Quantitative extraction revealed a significant decrease in lipid droplet content following OD treatment (p = 0.008).

### 3.7 Changes in extracellular vesicle numbers

Extracellular vesicles (EVs) are a heterogeneous population of membrane-bound structures secreted by cells into the extracellular space, playing key roles in intercellular communication, modulation of signaling pathways, and redistribution of biomolecules (Yu et al., 2024). Among the most relevant EV subtypes in the context of cell–cell communication are exosomes, which are formed through the exocytosis of multivesicular bodies (Arya et al., 2024). Their membranes differ from the plasma membrane in composition, being enriched in cholesterol [up to ∼80% of total lipids (Nishida-Aoki et al., 2020)] and sphingomyelin, reflecting their biogenesis and contributing to vesicle stabilization (Skotland et al., 2019). Although exosomes constitute only a small fraction of intracellular lipids, multivesicular bodies from which they originate contain up to 80% of cytosolic cholesterol (Möbius et al., 2003). Moreover, previous studies have shown that alterations in cellular lipid metabolism can directly influence EV secretion (Shkurnikov et al., 2024; Abdullah et al., 2021). Therefore, quantitative characteristics of EVs can be considered an indirect indicator of cellular metabolic status.

To assess the impact of hypoxia on EV secretion, a hypoxic environment was modeled using OD treatment. The EVs isolated in this study corresponded to the size range typical of exosomes (50–120 nm) (Supplementary Figures S8A,S8B) and exhibited a modal diameter of approximately 110 nm (Supplementary Figure S8C). Quantitative analysis revealed that OD treatment induced a statistically significant 2.8-fold increase in the number of secreted EVs compared with the control (p = 0.048) (Supplementary Figure S8D).

## 4 Discussion

Physiological oxygen tension in the placenta is approximately 2%–3% during the first trimester and 6%–8% during the second and third trimesters (Tuuli et al., 2011), with HIFα stabilization occurring already at 8% oxygen (Tache et al., 2013). Low oxygen levels play a key role in trophoblast development and differentiation: activation of the HIF signaling pathway promotes EVT lineage formation and supports progenitor cell survival (Chang et al., 2018). In preeclampsia, hypoxia is further exacerbated, likely due to impaired maternal blood flow and dysregulation of vasoconstriction by extravillous trophoblasts (Kumar et al., 2018). In our study, hypoxia pathway activation was observed across all trophoblast subpopulations and was accompanied by changes in the expression of genes involved in lipid metabolism. Single-cell RNA-seq data indicate that trophoblast populations—SCT, VCT, and EVT—differ both in baseline expression of lipid metabolism genes and in their response to pathological conditions such as preeclampsia.

The syncytiotrophoblast (SCT), which serves as the main interface between maternal blood and the fetus, shows the highest expression of genes involved in lipid transport, including *LDLR* and components of the clathrin complex (*CLTA*, *CLTB*). In preeclampsia, SCT exhibits reduced *LDLR* expression and alterations in clathrin-mediated endocytosis genes, accompanied by increased levels of *SCARB1* and *CAV1*. This may indicate a shift in lipid transport mechanisms, with reduced LDL uptake via LDLR and enhanced transcytosis via SR-BI (see Supplementary Figure S11). Such remodeling likely represents an adaptive mechanism to limit the uptake of oxidized LDL and Lp(a), which are elevated more than twofold in preeclampsia (Konrad et al., 2020; Wang et al., 1998). In our study, *ABCA1* mRNA expression was increased in all trophoblast subpopulations and in BeWo b30 cells under hypoxia, whereas previous reports have described decreased apical *ABCA1* protein expression in SCT during preeclampsia (Baumann et al., 2013). This discrepancy may reflect differences between transcriptomic and proteomic regulation and warrants further validation using additional single-cell datasets.

All trophoblast subpopulations showed decreased expression of cholesterol biosynthesis genes, most pronounced in VCT and SCT. Systemically, preeclampsia is associated with elevated cellular cholesterol levels (Lee et al., 2019)however, cholesterol-dependent signaling cascades (Wnt/β-catenin, Hedgehog, eNOS) are suppressed, consistent with a deficit of bioavailable cholesterol despite compensatory activation of its synthesis and efflux (Hart, 2024). Downregulation of cholesterol biosynthesis may have adaptive value, considering the high energy cost of this pathway (>11 O_2_ molecules and >100 ATP equivalents per product molecule (Ridgway and McLeod, 2008)) and the fact that cholesterol enrichment of membranes limits oxygen diffusion (Zuniga-Hertz and Patel, 2019). Thus, the combination of reduced synthesis, decreased endocytosis, and enhanced cholesterol efflux can be considered a cellular remodeling mechanism aimed at alleviating hypoxic stress. Increased *SCARB1* expression likely serves as a compensatory mechanism to maintain lipid transport to the fetus via transcytosis. These mechanisms may represent potential targets for interventions aimed at improving fetal lipid delivery in preeclampsia.

Interestingly, in EVT, where baseline *LDLR* expression is lower than in other trophoblast populations, preeclampsia was associated with an increase in *LDLR* expression. Concurrently, *SCARB1* and *ABCA1* expression increased, while *ABCG1* decreased, suggesting activation of mechanisms aimed at maintaining cholesterol transcytosis. However, it is known that preeclampsia is accompanied by a reduction in the number of EVT cells lining maternal vessels and directly contacting maternal blood, limiting nutrient and lipid delivery to the fetus and contributing to fetal growth restriction (Kumar et al., 2018; Meakin et al., 2022). The observed expression changes likely reflect a shortage of exogenous lipids, which is also consistent with the upregulation of the *GOBP_MEMBRANE_LIPID_CATABOLIC_PROCESS* pathway identified in our analysis.

Experiments with the BeWo b30 cell line treated with OD confirmed the key role of hypoxia in lipid metabolic remodeling. Under hypoxia, BeWo b30 cells exhibited an expression pattern most similar to SCT under preeclampsia. Specifically, genes involved in exogenous cholesterol uptake and lipid biosynthesis were downregulated, accompanied by increased expression of *SCARB1*, *CAV1*, and *ABCA1*. Simultaneously, expression of *SOAT1* and *CHP1* (FC = −1.53, padj = 1.03e-05), a recently identified regulator of lipid droplet size (Yang G. et al., 2025), was decreased, indicating reduced lipid storage. Single-cell data show that *SOAT1* expression in SCT is below detectable levels, consistent with previous reports of absent lipid droplet accumulation in this population (Kolahi et al., 2016). Thus, hypoxia acts as an inducer of metabolic remodeling, enhancing cholesterol transcytosis and efflux while reducing its storage, reproducing the alterations characteristic of SCT and EVT in preeclampsia. These results further support the applicability of BeWo b30 under hypoxic conditions as a model of SCT for studying lipid metabolism disruptions in preeclampsia (Zhao et al., 2021).

Recent data highlight the key role of *SNX17* and *PCSK9* in regulating lipoprotein transport in trophoblasts. In women with maternal supraphysiological hypercholesterolemia (MSPH), characterized by markedly elevated maternal cholesterol levels (Barrett et al., 2014; Fuenzalida et al., 2018), neonatal cholesterol concentrations remain within physiological ranges (Morales et al., 2025). This phenomenon has been linked to reduced LDLR recycling due to decreased *PCSK9* and *SNX17* expression in the presence of cholesterol excess (Morales et al., 2025). In preeclampsia, *PCSK9* expression has also been reported to decrease relative to controls, most prominently in early-onset disease (PE vs. CTRL, median: 0.2 vs. 0.9, p = 0.010; PE vs. LP, median: 0.2 vs. 1.2, p = 0.012) (Mennitti et al., 2024). Together with data showing higher *PCSK9* concentrations in fetal compared with maternal circulation (Vaught et al., 2023), this suggests that restriction of receptor recycling, rather than decreased expression, may be the primary mechanism protecting the fetus from excessive lipid uptake.

In the BeWo b30 preeclampsia model, *LDLR* expression remained unchanged, whereas *PCSK9* increased fivefold and *SNX17* decreased. Single-cell RNA-seq did not detect PCSK9 expression, likely due to methodological limitations and low sequencing depth. Data on the role of hypoxia in regulating *PCSK9* remain limited. For instance, in patients with obstructive sleep apnea, which involves chronic intermittent hypoxia, PCSK9 levels were higher than in control subjects (Reveyaz et al., 2025). Mechanistically, HIF-1α is known to activate SREBF (Liu et al., 2014), a transcription factor regulating lipid metabolism, including *PCSK9*. However, our transcriptomic analysis did not reveal statistically significant activation of factors regulating *PCSK9* expression. Thus, the mechanisms controlling *PCSK9* and *SNX17* under hypoxia and preeclampsia remain insufficiently understood and may have practical relevance for preventing fetal lipid imbalance and developing new therapeutic strategies in preeclampsia.

Currently, therapeutic options for preeclampsia remain limited, with low-dose aspirin being the only widely recommended prophylactic intervention, underscoring the urgent need for new mechanistically grounded approaches (Magee et al., 2022). At present, clinically approved therapeutics exist only for a subset of the lipid-regulatory proteins highlighted in our analysis. PCSK9 is the best-established target, with monoclonal antibodies and siRNA-based agents used to lower LDL cholesterol in hypercholesterolemia. However, several independent studies report that placental PCSK9 expression is reduced in preeclampsia (Mennitti et al., 2024; Vaught et al., 2023). These works suggest that PCSK9 may exert a protective role in trophoblasts by limiting cholesterol overload under conditions of increased maternal lipid levels during pregnancy. Indeed, lowering LDL cholesterol with statins has been shown to enhance angiogenesis, mitigate endothelial dysfunction characteristic of preeclampsia, and reduce inflammation, collectively ameliorating disease manifestations (Smith and Costantine, 2022). Thus, further suppression of PCSK9 through targeted therapies may have a dual effect: on the one hand reducing circulating cholesterol, but on the other potentially diminishing a trophoblast-protective mechanism. For ABCA1, pharmacological agonists such as CS-6253 have demonstrated preclinical efficacy in enhancing cholesterol efflux, although no agents targeting this transporter are approved for cardiovascular or obstetric use (Noveir et al., 2022). SCARB1 and SOAT1, despite evidence from metabolic disease models indicating that modulation of transcytosis or cholesterol esterification can alter intracellular lipid handling, currently lack any marketed therapeutics. Thus, while existing drugs targeting PCSK9 and ABCA1 show that components of these pathways are pharmacologically tractable, their potential utility in preeclampsia would differ from current symptom-oriented approaches by directly modulating placental lipid transport and metabolic adaptation to hypoxia. Nevertheless, translational applicability requires careful evaluation of safety, off-target effects and trophoblast-specific responses.

Exosomes secreted by the placenta and embryonic cells suppress the maternal immune system and promote pregnancy establishment, as well as fetal development and survival (de Lima Kaminski et al., 2019). Placental EVs also play a key role in regulating fetal lipid metabolism (Luo et al., 2025). However, in pregnancy pathologies such as preeclampsia, this process is disrupted. Patients with preeclampsia exhibit a significant increase in the number of exosomes released by the syncytiotrophoblast (Goswam et al., 2006). Hypoxia induction enhances exosome release from cytotrophoblasts and increases their bioactivity (Salomon et al., 2013). Our results show that, despite the downregulation of genes involved in cholesterol synthesis and transport, a key lipid in exosome biogenesis, hypoxia induced by the oxiquinoline derivative also increases vesicle exocytosis. This is particularly interesting, as previous studies demonstrated that exosomes from umbilical cord plasma can suppress *HMGCS1*, thereby inhibiting cholesterol synthesis and ultimately inducing endothelial dysfunction (Ying et al., 2021). In a recent study, placental hypoxia was shown to promote the release of EVs enriched in lipid peroxidation products, and their exposure similarly leads to endothelial dysfunction characteristic of preeclampsia (Park et al., 2025). Another study demonstrated that hypoxia increases expression of Hepatocyte Growth Factor-regulated Tyrosine Kinase Substrate (HGS), which appears to regulate vesicle secretion and markedly alter their cargo (Wang et al., 2025). However, in our BeWo cell model, HGS expression did not change significantly under OD treatment (p = 0.54).

A key constraint of our study is the reliance on a single publicly available single-cell RNA-seq dataset and the lack of detailed clinical information regarding patient therapies. Additionally, we employed only one trophoblast cell model, BeWo b30; while this line is widely used in hypoxia-related trophoblast research, future studies would benefit from including additional trophoblast cell lines to validate the observed effects more broadly. A limitation of our study is the lack of analysis of exosome cargo composition, which warrants further investigation. The data suggest that the relationship between HIF1A-mediated hypoxia and exosome secretion in trophoblasts may be regulated by an alternative, yet unidentified mechanism. In light of the recently demonstrated role of exosomes in lipid delivery (Palmulli et al., 2024), hypoxia-induced enhancement of their secretion likely impacts trophoblast lipid metabolism and may contribute to disease pathogenesis. Finally, although our transcriptomic analyses provide insight into the regulation of cholesterol uptake, transcytosis, and efflux pathways, functional lipid secretion was not directly assessed. Future studies incorporating targeted lipidomic profiling and direct evaluation of lipoprotein secretion will be essential to clarify how hypoxia and preeclampsia alter trophoblast lipid homeostasis at the metabolic level.

## 5 Conclusion

Our data demonstrate that under preeclampsia and hypoxic stress, trophoblast cells exhibit substantial changes in the expression of genes involved in lipid metabolism. Distinct trophoblast subpopulations (SCT, VCT, EVT) show transcriptional alterations consistent with reduced LDL-mediated endocytosis, enhanced cholesterol transcytosis via SCARB1, increased cholesterol efflux, and remodeling of cholesterol biosynthesis pathways. It should be noted that these observations are limited to gene expression changes and do not directly assess functional activity. Nevertheless, the coordinated transcriptional patterns suggest a potential reprogramming of lipid handling that may contribute to maintaining fetal lipid supply and responding to hypoxic stress.

Importantly, our findings provide insight into the molecular basis of lipid metabolism dysregulation in preeclampsia and highlight potential targets for intervention. By identifying key regulators of lipid delivery to the fetus and cellular responses to hypoxic stress—such as *PCSK9*, *SNX17*, *SCARB1*, *ABCA1*, and *SOAT1*—this study may inform the development of novel preventive and therapeutic strategies to mitigate pregnancy complications associated with abnormal lipid homeostasis. Furthermore, the observed effects in the BeWo b30 hypoxia model support its relevance as a tool for studying placental lipid metabolism under pathological conditions.

## Data Availability

The datasets presented in this study can be found in online repositories. The names of the repository/repositories and accession number(s) can be found in the article/Supplementary Material.
