# Supplementary material for: Comparative analysis of lipid metabolism in trophoblast subpopulations in preeclampsia and in vitro hypoxia model
Source: Front Mol Biosci. 2025 Dec 9;12:1731126. doi: 10.3389/fmolb.2025.1731126 (PMC12722821; doi:10.3389/fmolb.2025.1731126)
Supplement: Supplementary file 3 [file Supplementaryfile1.pdf]

Supplementary materials

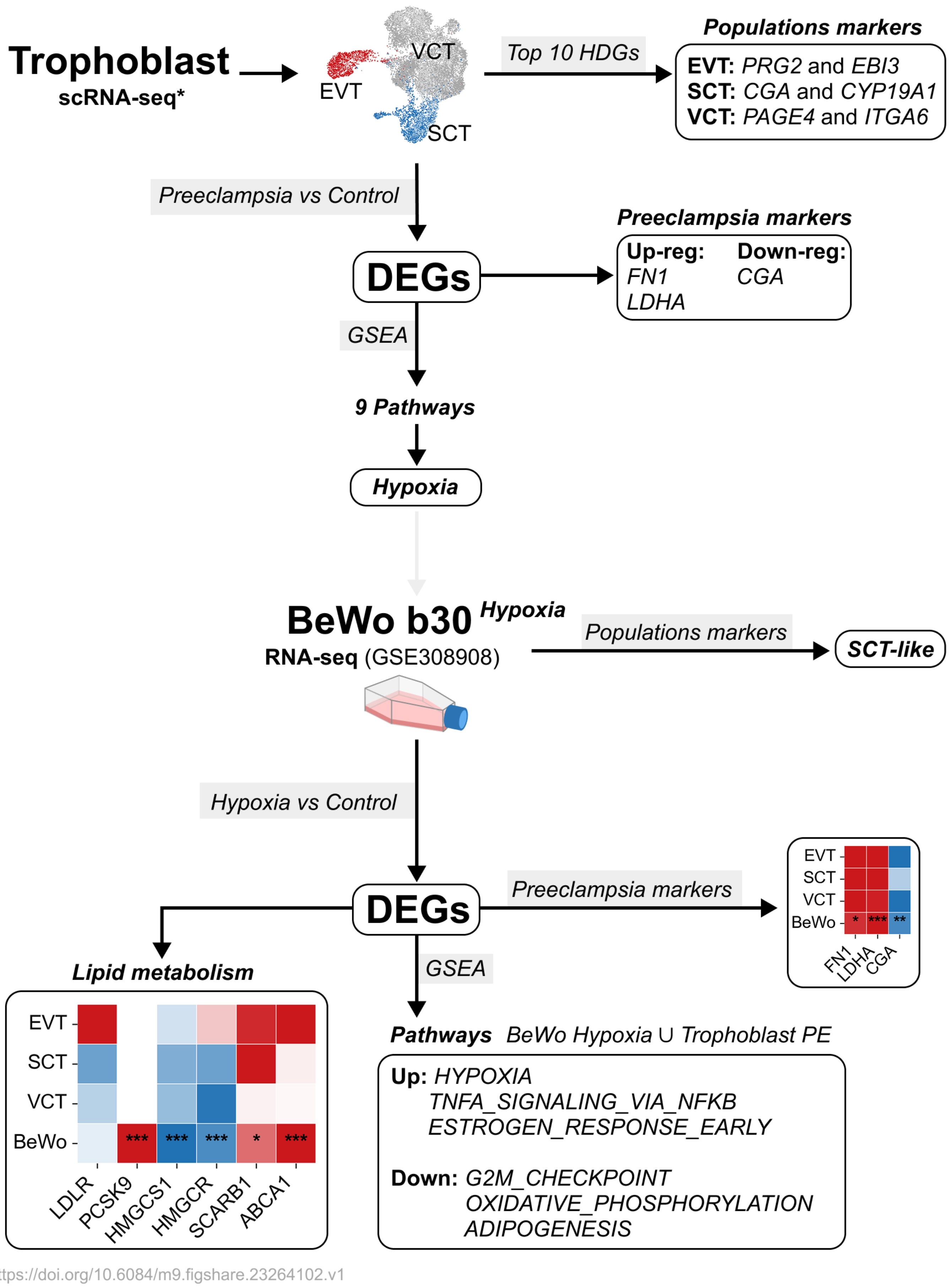

Figure S1. Schematic overview of the study design.

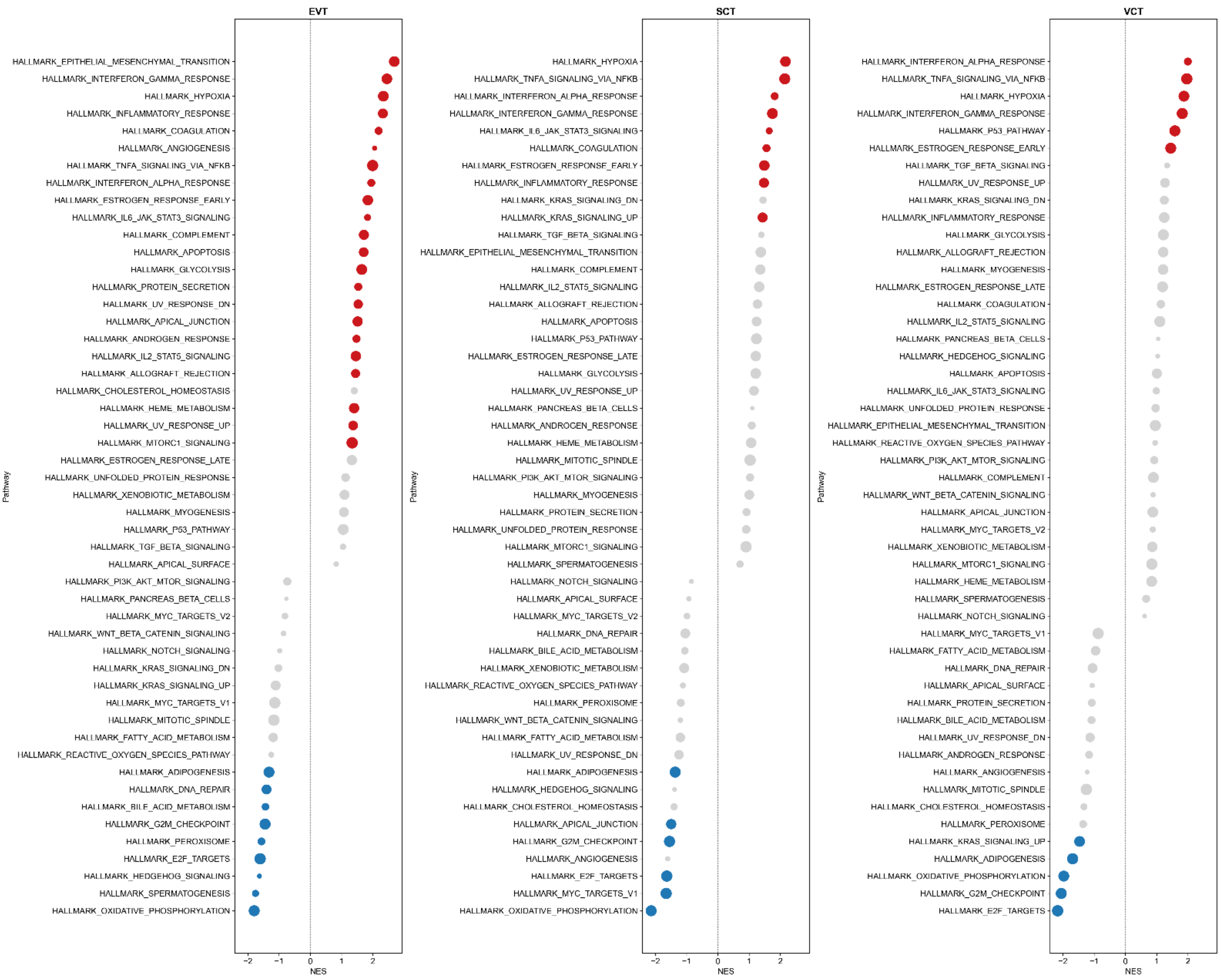

Figure S2. GSEA results showing enrichment of Hallmark pathways in trophoblast subpopulations (EVT, SCT, VCT) in preeclampsia compared to control. Red indicates significantly upregulated pathways (padj < 0.05), blue indicates significantly downregulated pathways (padj < 0.05), and grey indicates pathways without significant changes.

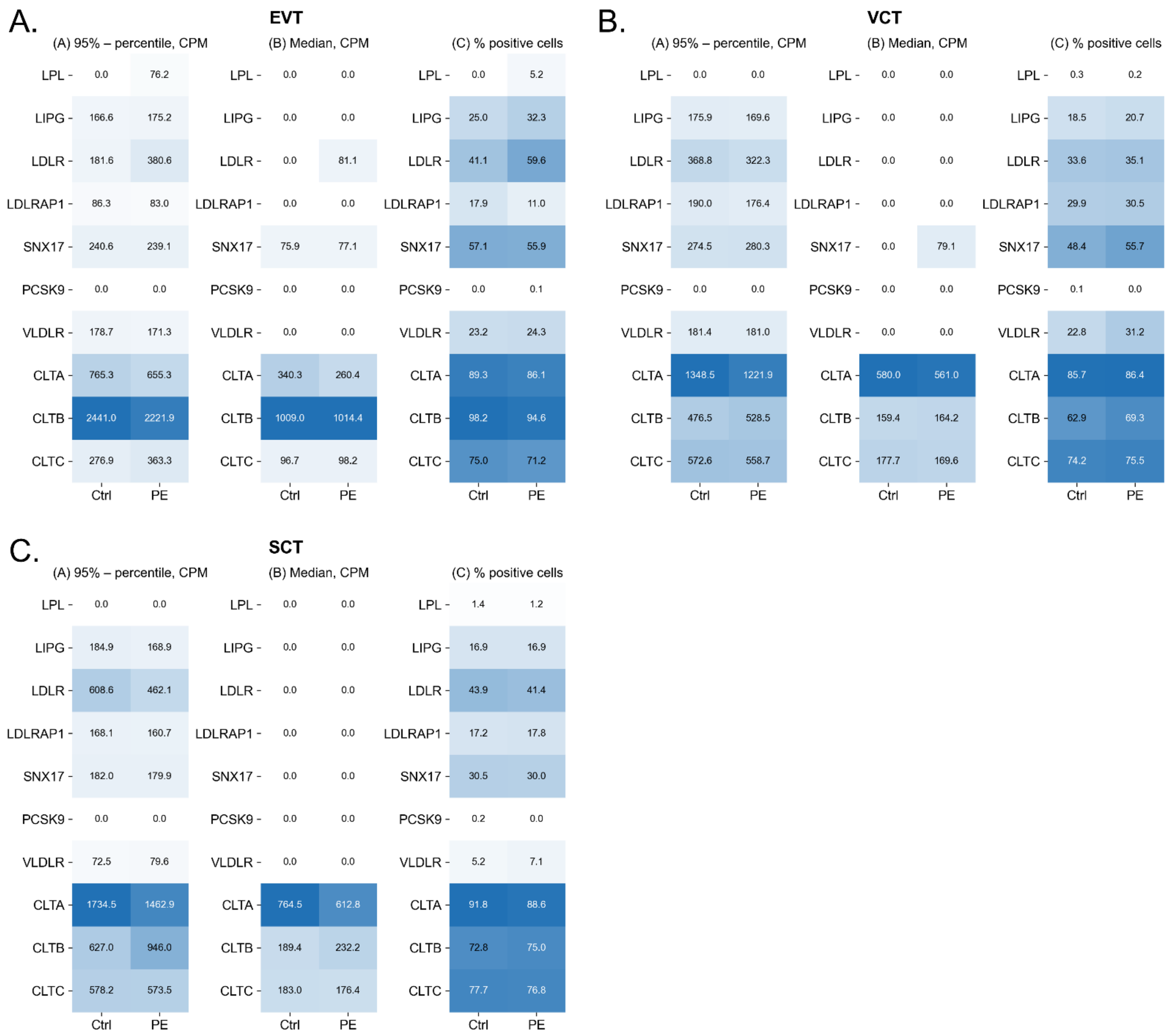

Figure S3. Single-cell RNA sequencing data reveal differences in the expression of genes associated with lipid uptake between control (Ctrl) and preeclampsia (PE) trophoblast populations. A.- EVT; B.- VCT; C.- SCT; Panels show: (A) 95th percentile of expression (CPM), (B) median expression (CPM), and (C) proportion of positive cells (%).

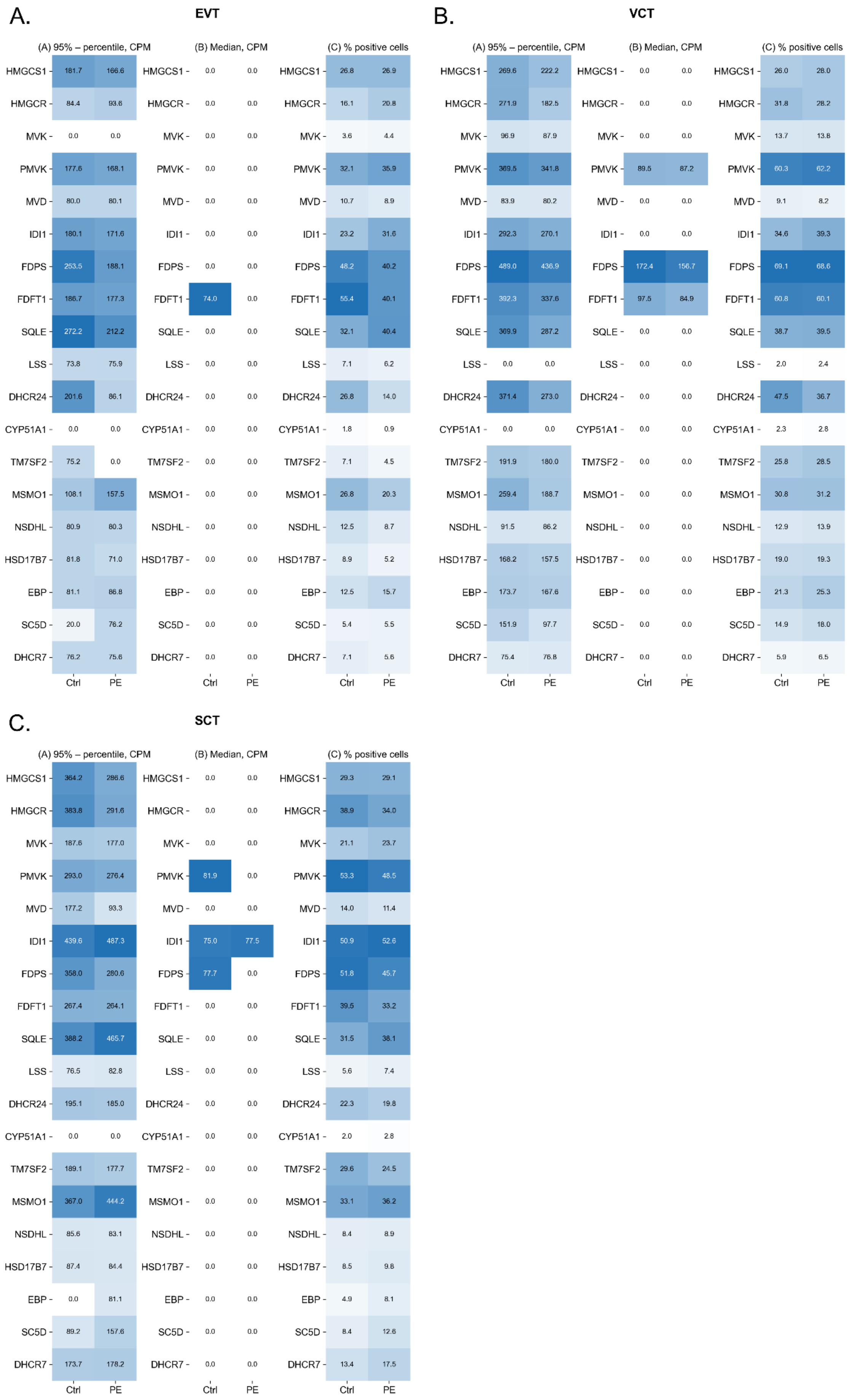

Figure S4. Single-cell RNA sequencing data reveal differences in the expression of genes associated with cholesterol synthesis between control (Ctrl) and preeclampsia (PE) trophoblast populations. A.- EVT; B.- VCT; C.- SCT; Panels show: (A) 95th percentile of expression (CPM), (B) median expression (CPM), and (C) proportion of positive cells (%).

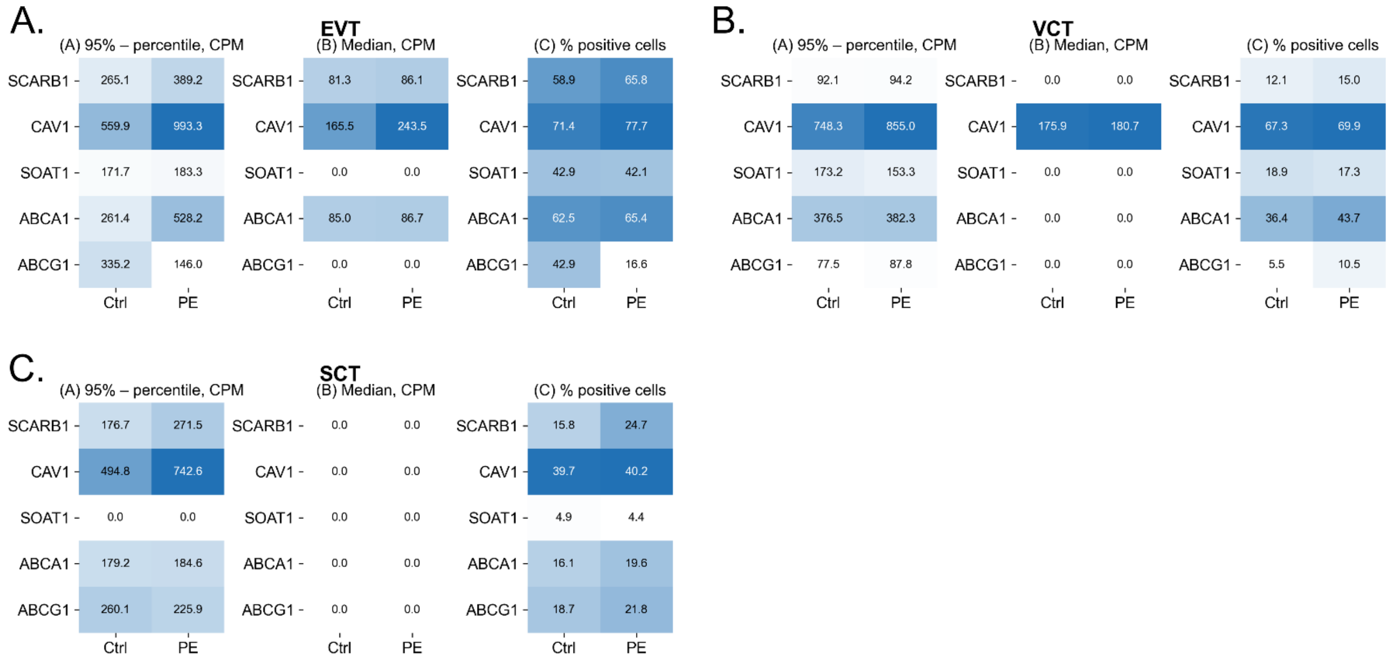

Figure S5. Single-cell RNA sequencing data reveal differences in the expression of genes associated with lipid transcytosis and efflux between control (Ctrl) and preeclampsia (PE) trophoblast populations. A.- EVT; B.- VCT; C.- SCT; Panels show: (A) 95th percentile of expression (CPM), (B) median expression (CPM), and (C) proportion of positive cells (%).

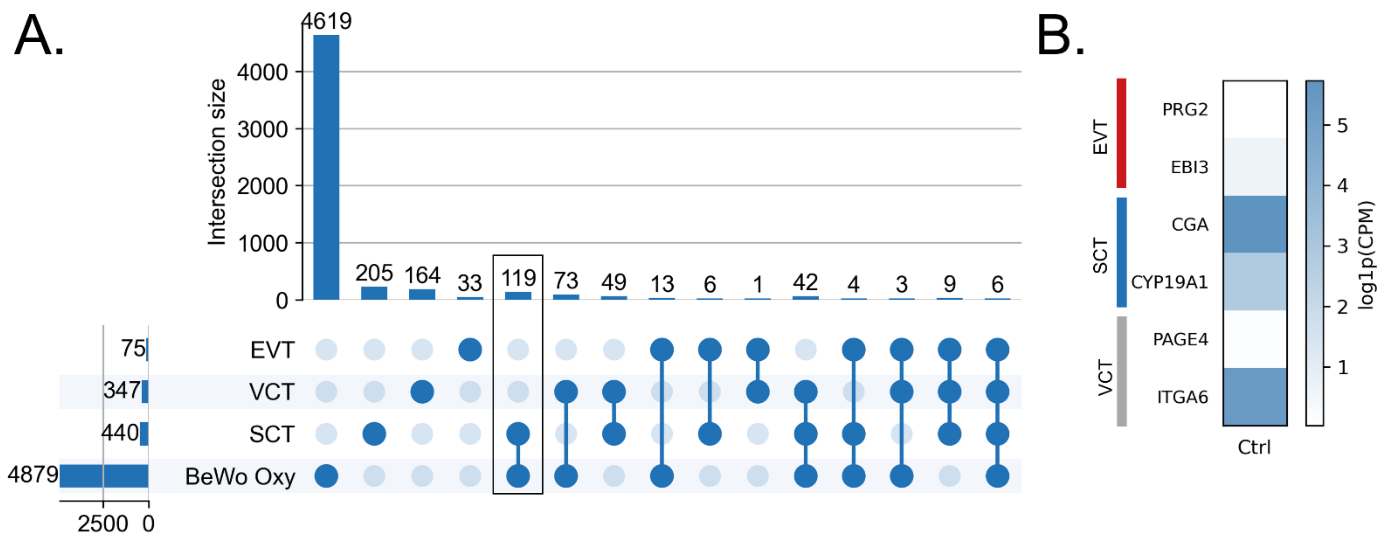

Figure S6. A. UpSet plot showing the overlap of differentially expressed genes (DEGs) between BeWo b30 cells treated with oxyquinoline derivatives and trophoblast subpopulations from preeclamptic placenta. B. Expression of trophoblast marker genes in BeWo b30 cells, log1p(CPM).

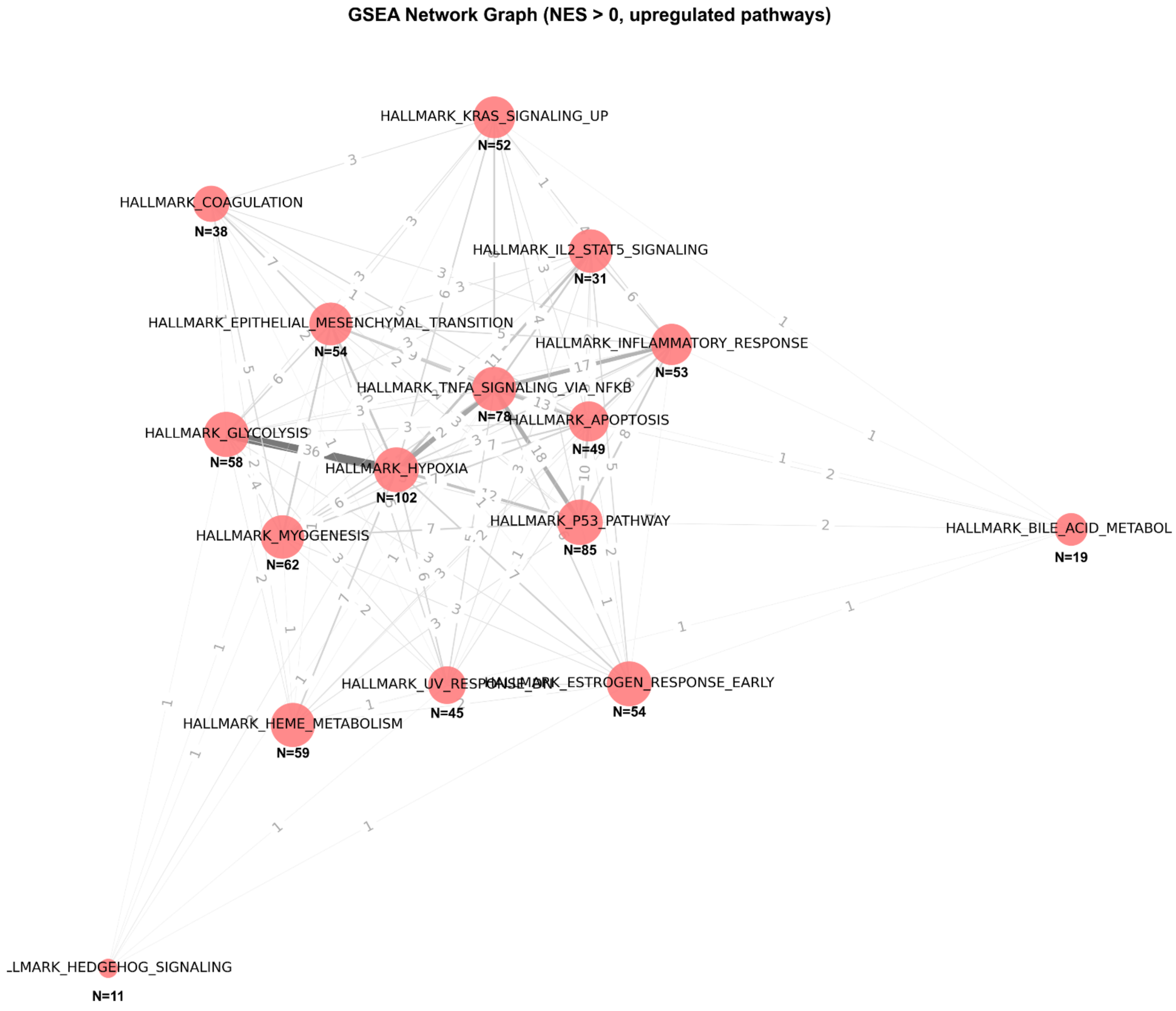

Figure S7. Schematic network illustrating the overlap of genes among pathways upregulated (NES > 0) following hypoxia induced by oxyquinoline derivatives in BeWo b30 cells. Each node represents a significantly enriched pathway (*padj* < 0.05), with node size proportional to the number of genes in the leading edge. Edge thickness reflects the number of shared genes between pathways.

GSEA Network Graph (NES < 0, downregulated pathways)

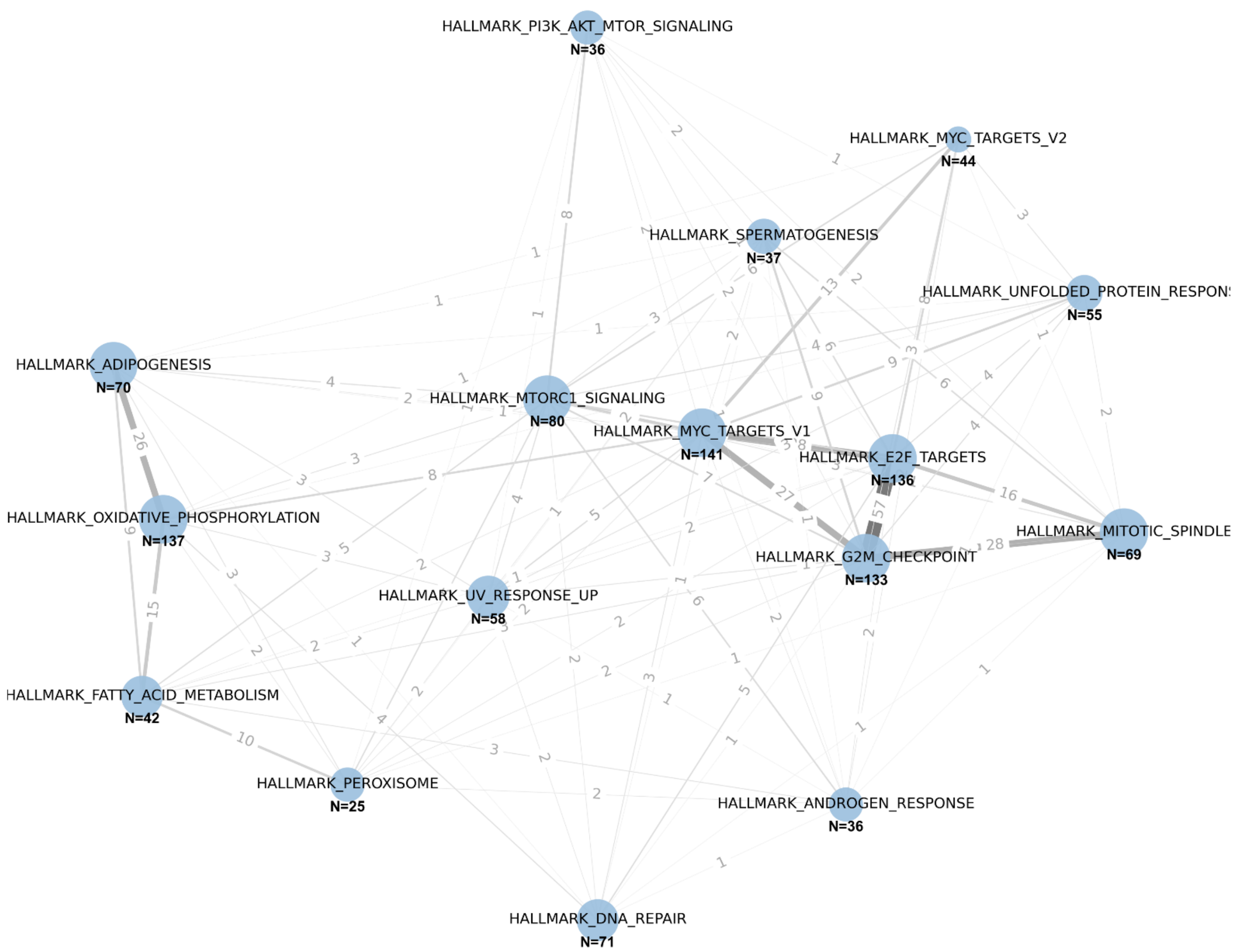

Figure S8. Schematic network illustrating the overlap of genes among pathways upregulated (NES < 0) following hypoxia induced by oxyquinoline derivatives in BeWo b30 cells. Each node represents a significantly enriched pathway (*padj* < 0.05), with node size proportional to the number of genes in the leading edge. Edge thickness reflects the number of shared genes between pathways.

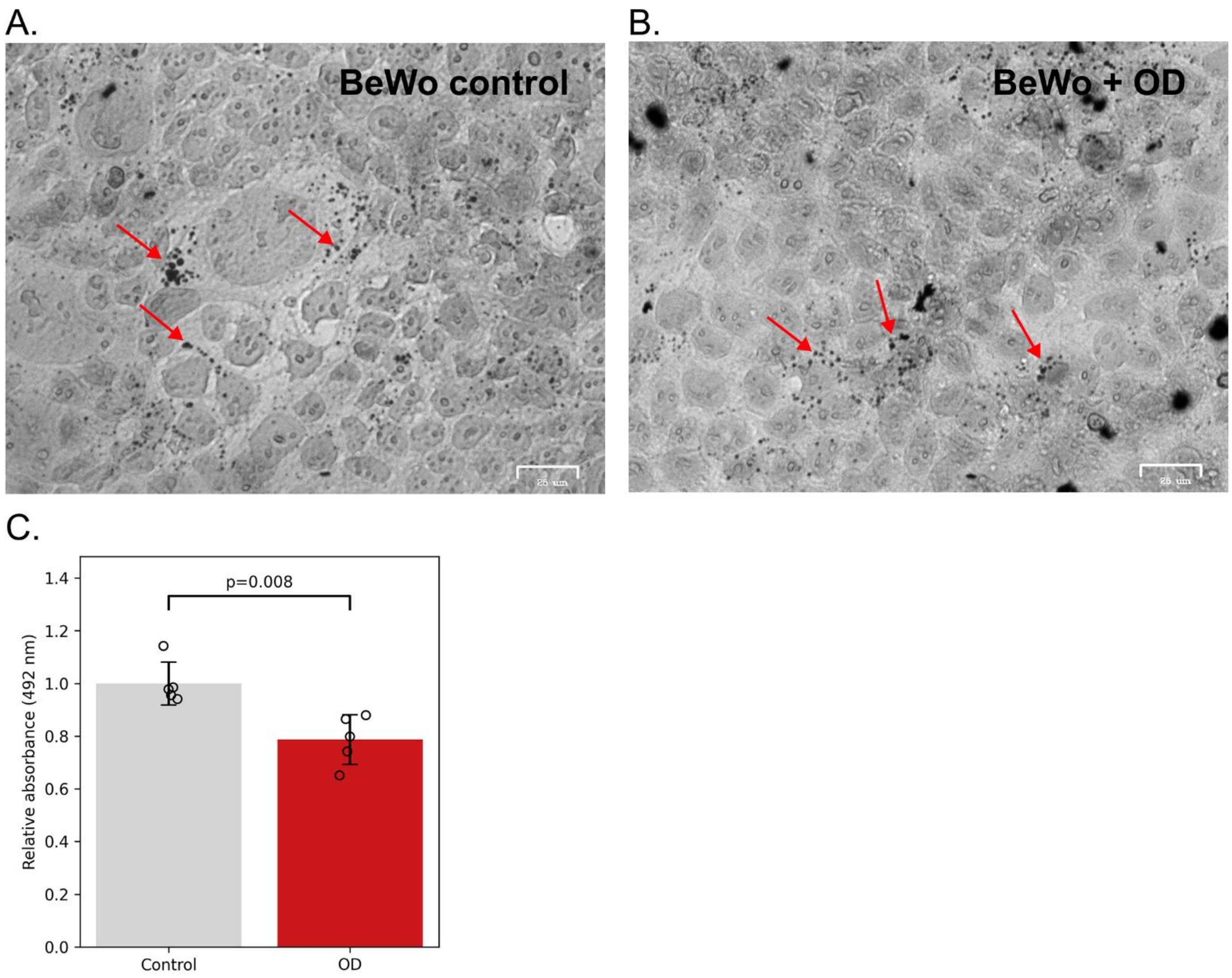

Figure S9. Results of Oil Red O staining in BeWo b30 cells. A. Microscopic image of control cells. B. Cells after treatment with an oxyquinoline derivative. C. Relative absorbance at 492 nm following dye extraction, showing reduced lipid accumulation after treatment.

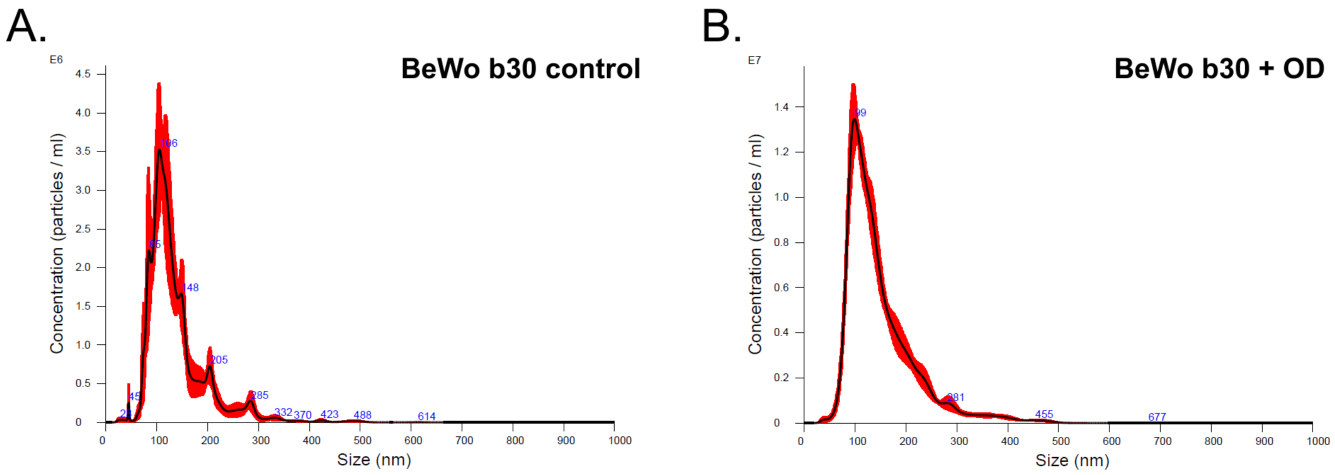

C.

	Control		+ OD	
	Mean	+/- std	Mean	+/- std
**Mean** (nm)	155.4	21.9	153.9	7.6
**Mode** (nm)	113.6	8.7	111.0	12.1
**Concentration** (part./ml)	3.1×10^8	8.4×10^7	8.7×10^8	3.4×10^8

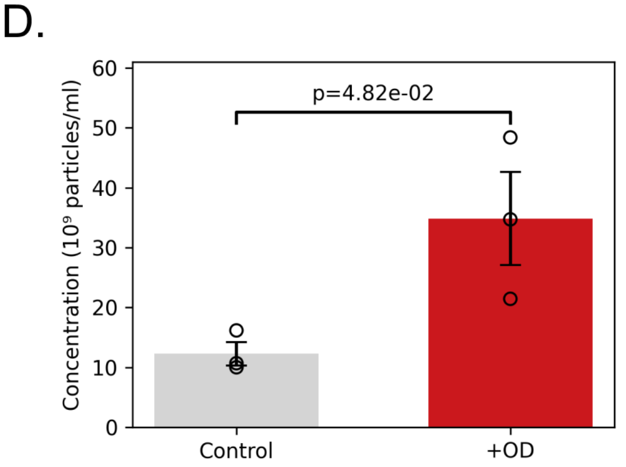

Figure S10. Characterization of small extracellular vesicles (sEVs) isolated from BeWo b30 cell culture medium. A. Size distribution of particles isolated from the culture medium of control cells (five measurements). B. Size distribution of particles isolated after hypoxia induction (five measurements). C. Summary of particle size and concentration across three biological replicates. D. Boxplot showing the concentration of sEVs obtained from three biological replicates of control cells and cells treated with an oxyquinoline derivative (+OD).

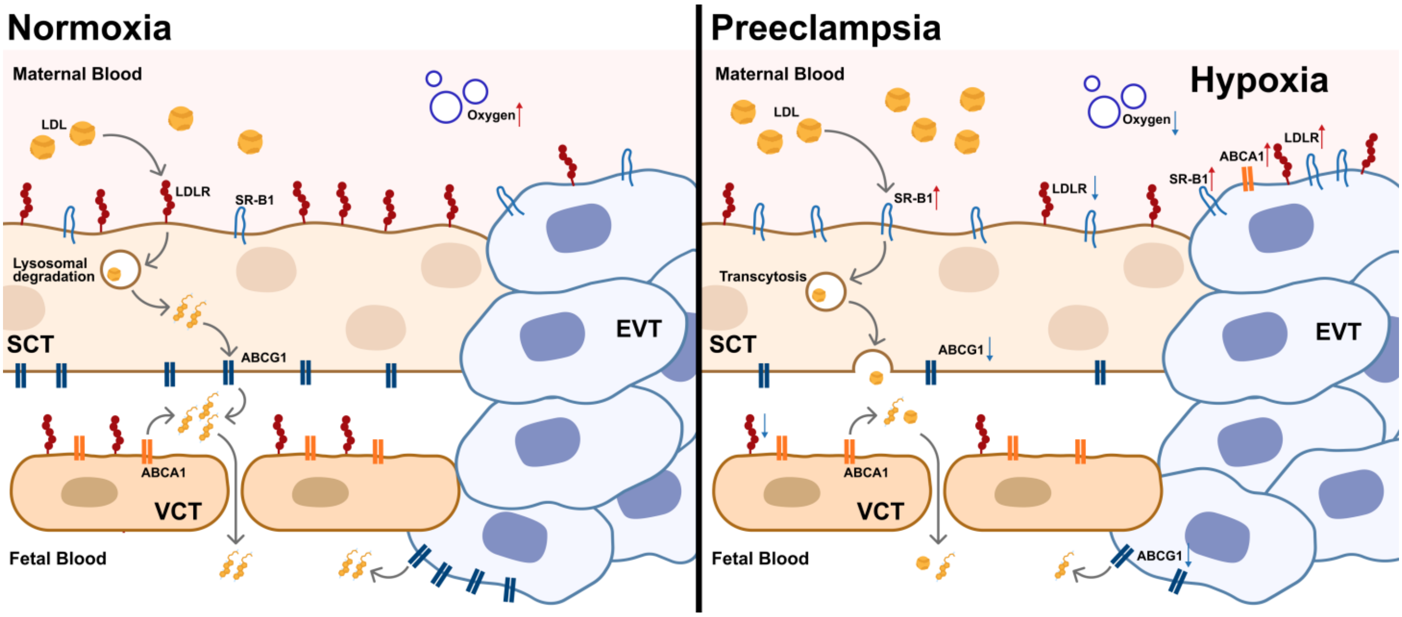

Figure S11. Graphical summary illustrating the observed changes in gene expression across trophoblast subpopulations in preeclampsia. Normoxia – left, Preeclampsia – right: syncytiotrophoblast (SCT), cytotrophoblast (VCT), and extravillous trophoblast (EVT).

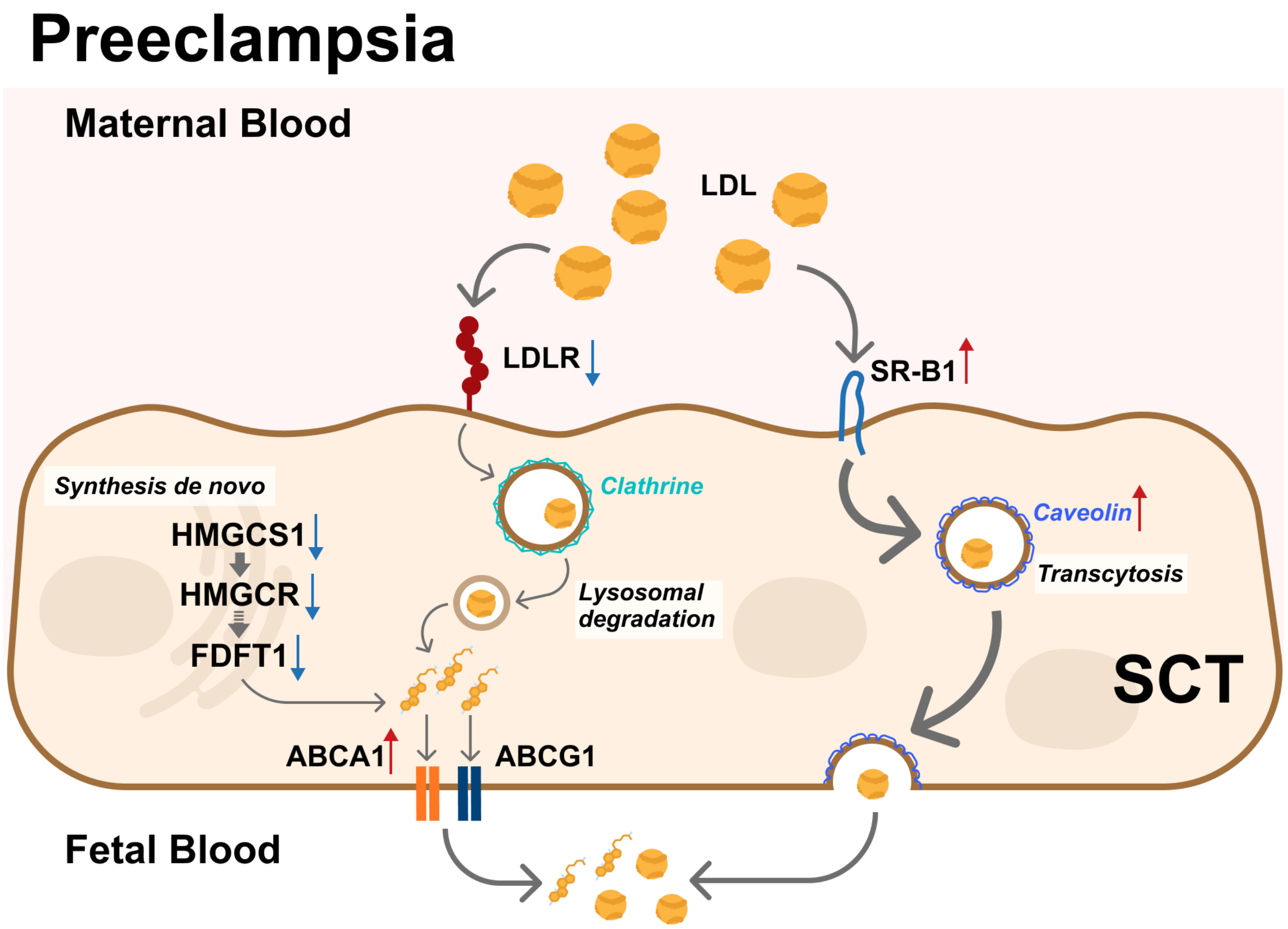

Figure S12. Graphical abstract illustrating the observed changes in gene expression in the syncytiotrophoblast (SCT), confirmed in the hypoxia BeWo B30 model, during preeclampsia.
